# Development of trastuzumab-resistant human gastric carcinoma cell lines and mechanisms of drug resistance

**DOI:** 10.1038/srep11634

**Published:** 2015-06-25

**Authors:** Qiang Zuo, Jing Liu, Jingwen Zhang, Mengwan Wu, Lihong Guo, Wangjun Liao

**Affiliations:** 1 Department of Oncology, Nanfang Hospital, Southern Medical University, Guangzhou, Guangdong Province, China

## Abstract

Trastuzumab has been successfully employed for the treatment of Her-2-positive gastric cancer. However, there are problems with both primary and secondary resistance to trastuzumab. In this study, we employed the human gastric carcinoma cell line NCI-N87 with high Her-2 expression to create trastuzumab-resistant NCI-N87/TR cells by stepwise exposure to increasing doses of trastuzumab. Western blotting and Real-time PCR were conducted to detect protein and gene levels. Compared with NCI-N87 cells, the expression of P-IGF-1R and P-AKT proteins was significantly increased in NCI-N87/TR cells (both *P* = 0.000), while PTEN gene and protein expression showed a significant decrease (both *P* = 0.000). In addition, mutations of the PTEN gene were detected at exons 5, 7, and 8. The sensitivity of NCI-N87/TR cells to trastuzumab was increased by transfection with the PTEN gene, or by incubation with a PI3K inhibitor (LY294002) or an IGF-IR inhibitor (AG1024), as well as siRNA targeting PI3K p110 or IGF-1R. Taken together, our findings showed that activation of the PI3K-AKT signaling pathway was one of the major mechanisms leading to resistance of NCI-N87/TR gastric cancer cells to trastuzumab, which was probably associated with PTEN gene down-regulation and mutation, as well as with over-activity of the IGF-1R signaling pathway.

The human epidermal growth factor receptor (HER) family consists of 4 members (Her-1/EGFR, Her-2/neu, Her-3, and Her-4). The Her-2/neu gene is located on chromosome 17q21 and the protein tyrosine kinase (PTK) activation is observed in the intracellular domain. When ligands bind with the extracellular ligand-binding domain of Her-2 protein, a cascade of signal transduction is induced, including activation of PTK, the Ras-Raf-MAPK signaling pathway, and the PI3K-AKT-mTOR signaling pathway, resulting in effects on various cellular processes, including proliferation, apoptosis, adhesion, migration, and differentiation^1^,^2^.

Over-expression of Her-2 protein is found in various tumor cells, and the signal transduction pathways it mediates have a close connection with carcinogenesis and with tumor progression and the prognosis. Her-2 over-expression is also found in gastric cancer, and is associated with a poor prognosis^3^. Trastuzumab (Herceptin) is a humanized monoclonal antibody that targets Her-2 recombinant DNA, and inhibits cell growth by antagonizing the Her-2 signal transduction pathway^4^. Trastuzumab has already been successfully employed for the treatment of metastatic breast cancer and gastric cancer with Her-2 over-expression, and has achieved a promising therapeutic effect^5^,^6^,^7^. However, primary and secondary resistance to trastuzumab have severely influenced its clinical application.

As is well known, signal transduction pathways are multi-factor, multi-link, and cross-talking network systems, so multiple factors influence resistance to molecular-targeting drugs. Previous studies on trastuzumab resistance have mainly focused on breast cancer^8^,^9^,^10^,^11^. However, the mechanisms of resistance to trastuzumab in gastric cancer remain unclear, and there have been few reports published.

In this study, we employed the human gastric cancer cell NCI-N87 with high expression of Her-2 protein to create a trastuzumab-resistant cell line (NCI-N87/TR) for the first time, analyzed the characteristics of NCI-N87/TR cells and investigated the detailed mechanisms regulating the *in vitro* resistance of gastric cancer to trastuzumab.

## Materials and Methods

### Materials

Human gastric cancer cells, including SGC7901, MKN45, NCI-N87 and MKN28, were obtained from the American Type Culture Collection (ATCC). Antibodies directed against Her-2, PTEN, EGFR/P-EGFR, IGF-1R/P-IGF-1R, AKT/P-AKT, survivin, cdk2, and p27kipl proteins were obtained from Abcam, while β-actin and α-tubulin antibodies were obtained from Boster and Sigma, respectively. A PI3K inhibitor (LY294002) and an IGF-1R inhibitor (AG1024) were purchased from Selleck, while the Trizol kit, pBabe-puro expression vector and liposome Lipofectamine were products of Invitrogen. Trastuzumab was provided by Roche Company (Shanghai), while 5-fluorouracil (5-FU), cisplatin (DDP), and paclitaxel (Taxol) were from Tianjin Pharmacy Company, Qilu Pharmacy Company, and Squibb Company, respectively. Finally, 3-(4, 5-dimethylthiazol-2-yl)-2, 5-diphenyl tetrazolium bromide (MTT) and RPMI-1640 culture medium were products of Sigma Company (USA).

### Induction of trastuzumab-resistant NCI-N87/TR cells

Aliquots of NCI-N87 cells in the exponential growth phase were seeded into 25 cm^2^ culture bottles. Trastuzumab (12 μg/ml) was added for 48 h during the mitotic phase, and then the cells were transferred into drug-free culture medium until the next mitotic phase (around 10 d), after which trastuzumab was added for the next 48 h at twice the previous concentration. We continued this process while observing cell death every day, changing to fresh complete culture medium, and performing the MTT assay regularly. This process was continued until the concentration of trastuzumab in the medium reached 3500 μg/ml after 150 days. Thus, NCI-N87 cells were obtained that grew stably in trastuzumab (3500 μg/ml)-containing medium, and these trastuzumab-resistant cells were named NCI-N87/TR cells.

### Resistance index (RI) and cross resistance via MTT assay

Cells in the exponential phase of growth were inoculated into each well of a 96-well plate at a density of 3 × 10^3^ cells per well, with three wells for each set of conditions. Cells were exposed to drugs at different concentrations for 48 h. Then MTT was then added to the wells at 5 mg/ml (20 μl per well), and the cells were incubated at 37 °C under 5% CO_2_ for 4 h. After carefully aspirating the medium, 150 μl of DMSO was added to each well to dissolve the Formazan crystals. Then a Bio-Tek microplate reader was used to measure the optical density (OD) at a wavelength of 490 nm. Cell viability was calculated according to the following equation: (drug-supplemented OD-blank control OD)/ (normal control OD-blank control OD) × 100%. Origin 6.1 software was utilized to plot the survival versus drug concentration curve and calculate the 50% inhibitory concentration (IC_50_). The resistance index (RI) was calculated as the ratio between the IC_50_ value of NCI-N87/TR cells and that of NCI-N87 cells.

### Detection of apoptosis

Apoptosis was detected with an AV/PI Double-Dye Apoptosis Kit according to the supplier’s directions. The cells were inoculated into a 60 mm culture dish and incubated for 24 h, followed by transfer to fresh culture medium and culture in an incubator. Cells were digested after 12 h of incubation to obtain a single-cell suspension, which was centrifuged at 1500 rpm for 3 min. After the supernatant was removed, the cells were washed twice with 1XPBS and centrifuged at 1500 rpm for 3 min. Then the supernatant was removed again, 500 μl of binding buffer was added, and the cells were cryopreserved. Next, 5 μl of Annexin V-FITC was added to the cell suspension and mixed thoroughly. Finally, 5 μl of PI was added and incubation was done for 5–15 min. The cells were subjected to flow cytometry 1 h later.

### Cell cycle analysis by flow cytometry (FCM)

Cells in the exponential growth phase were fixed by dropwise addition of 700 μl of precooled anhydrous alcohol at 4 °C overnight in the dark. Then the cells were suspended in 500 μl of RNase A (100 u/ml)-containing PBS buffer at 37 °C in the dark for 30 min and PI (2 mg/ml) was added in the dark over 30 min to a final concentration of 50 μg/mL. FCM was performed at 488 nm (Ex) and 525 nm (Em), and the cell proliferation index (CPI) was calculated by the formula CPI = (S + G2/M)/(G0/G1 + S + G2/M).

### Western blotting

Cells adherent to the plate were removed after the addition of PBS (0.5 ml) and were lysed in lysis buffer for 30 min. Centrifugation was performed, and the protein-containing supernatant was retained. Total protein (30 μg) and 5 × SDS loading buffer were mixed and boiled at 100 °C for 5 min. Then SDS-PAGE was performed with a separation gel (10%) at 100 V for 70 min and a stacking gel (4%) at 60 V for 30 min, followed by transfer of the proteins to membranes. Each membrane was blocked by incubation in TBST containing 5% skim milk powder for 1 h, followed by incubation with the primary antibody at 4 °C overnight and incubation with the secondary antibody at room temperature for 120 min. ECL detection was performed, and the film was processed immediately after exposure in a darkroom for 10 s to 10 min using Kodak developer and fixative. A PQ image acquisition and analysis system was employed to analyze the processed film.

### Real-time PCR

Total RNA was extracted using a Trizol kit, and the concentration and purity of the recovered RNA were measured. Then cDNA was synthesized from total RNA (2 ng to 2 μg) using MMLV-RT reverse transcriptase in accordance with the manufacturer’s instructions. Primers were supplied by Invitrogen, and their sequences are listed in Table 1.

The reaction mixture for real-time PCR was prepared in accordance with the specifications. The reaction system (total volume of 20 μl) included 2 × Mix SYBR Green I fluorescence reaction solution (10 μl), upstream and downstream primers (0.25 μl each), and templates of samples (1 μl). PCR was done for 40 cycles, with fluorescence signals being collected at the end of each extension step, and then the amplification curves were plotted. After 40 PCR cycles were completed, annealing was conducted (95 °C for 15 s, 60 °C for 30 s, and 95 °C for 15 s), and fluorescence signals were collected as the temperature was increased from 60 °C to 95 °C. Using this data, the melting curves were plotted.

### Sequencing of the PTEN gene

Sequencing was performed to detect mutations of the PTEN gene at exons 5, 7, and 8 in NCI-N87/TR and NCI-N87 cells. The procedure included the extraction of total RNA, reverse transcription for cDNA synthesis, and routine PCR. Primers were obtained from Invitrogen, and the primer sequences are listed in Table 2. PCR products were sent to Invitrogen for sequencing after gel extraction and purification.

### PTEN gene transfection

The target sequence of the PTEN gene was 1230 bp in length and the following primers were used to carry out PCR amplification (primer 1: Homo-PTEN-F-BamHI-F-1230: 5′CGCGGATCCATGACAGCCATCATCAAAGAG3′ and primer 2: Homo-PTEN-R-EcoRI-R-1230: 5′CCGGAATTCTCAGACTTTTGTAATTTGTGTATGC3′). Construction of the PTEN gene expression vector involved cutting the gel extraction DNA of the PTEN gene and the pBabe-puro vector with *BamH* I and *EcoR* I, respectively, followed by ligation with T4 DNA ligase (TaKaRa), and transformation of E. coli strain DH5α. After identification by colony PCR, screening was done by digestion using *BamH* I and *EcoR* I to confirm that PTEN had been inserted into the pBabe-puro vector. Finally, positive clones were sequenced and the results confirmed that the appropriate gene sequences were inserted.

Lipofectamine was used for transfection according to the manufacturer’s specifications. Briefly, NCI-N87/TR cells were inoculated into 6-well plates at a density of 3 × 10^5^ cells per well and incubated for 24 h, after which the medium was changed to opti-MEM for 2–6 h. The recombinant plasmid (4 μg) and Lipofectamine 2000 (10 μl) were diluted with Opti-MEM (250 μl) at room temperature for 5 min, after which the plasmid and Lipofectamine 2000 were mixed and let stand for 20 min. The mixture was added to the cells for transfection with incubation at 37 °C under 5% CO_2_ for 6 h, after which the medium was changed to complete medium and incubation was continued for 48 h before harvesting cellular proteins.

The cells were divided into 4 groups: NCI-N87 + vector, NCI-N87 + pBabe-puro-PTEN, NCI-N87/TR + vector, and NCI-N87/TR + pBabe-puro-PTEN. Western blotting was conducted to detect the levels of PTEN, AKT, and P-AKT proteins (the detailed procedure was explained above). The MTT assay was employed to detect the OD after cells were treated with trastuzumab at different concentrations, and IC_50_ values were calculated (the detailed procedures were explained above).

### Investigation of the PI3K-AKT signaling pathway

NCI-N87 and NCI-N87/TR cells were incubated with the PI3K inhibitor LY294002 at concentrations of 1.0 μM, 1.4 μM, and 1.8 μM. The cells were divided into 4 groups, which were NCI-N87, NCI-N87 + LY294002, NCI-N87/TR, and NCI-N87/TR + LY294002. The appropriate target sequences for PI3Kp110 gene interference included 5′CATGCCAGTGTGTGAATT3′. Lipofectamine was used for cell transfection with the guidance of the kit manufacturer’s specification. The cells were divided into 4 groups: NCI-N87, NCI-N87 + siPI3Kp110, NCI-N87/TR, and NCI-N87/TR + siPI3Kp110. Western blotting was conducted to detect AKT and P-AKT protein. The MTT assay was done to determine the OD for each group of cells and IC_50_ values were calculated.

### Study on the IGF-1R signaling pathway

NCI-N87 and NCI-N87/TR cells were incubated with the IGF-1R inhibitor AG1024 at concentrations of 0.1 μM, 0.2 μM, and 0.4 μM. The cells were divided into 4 groups, which were NCI-N87, NCI-N87 + AG1024, NCI-N87/TR, and NCI-N87/TR + AG1024. The appropriate target sequences for IGF-1R gene interference included 5′CACCATCTTCAAGGGCAA 3′. Lipofectamine was used for cell transfection with the guidance of the kit manufacturer’s specification. The cells were divided into 4 groups: NCI-N87, NCI-N87 + siIGF-1R, NCI-N87/TR, and NCI-N87/TR + siIGF-1R. Western blotting was performed to detect IGF-1R, P-IGF-1R, AKT, and P-AKT proteins. The MTT assay conducted to determine the OD for each group of cells and IC_50_ values were calculated.

### Statistical analysis

All data was represented as the mean of at least triplicate samples ± standard deviation. Statistical analysis included One-way ANOVA or Student’s t test using SPSS 13.0. *P* values less than 0.05 were considered statistically significant.

## Results

### Induction of trastuzumab resistance in NCI-N87/TR cells

**Figure 1:**
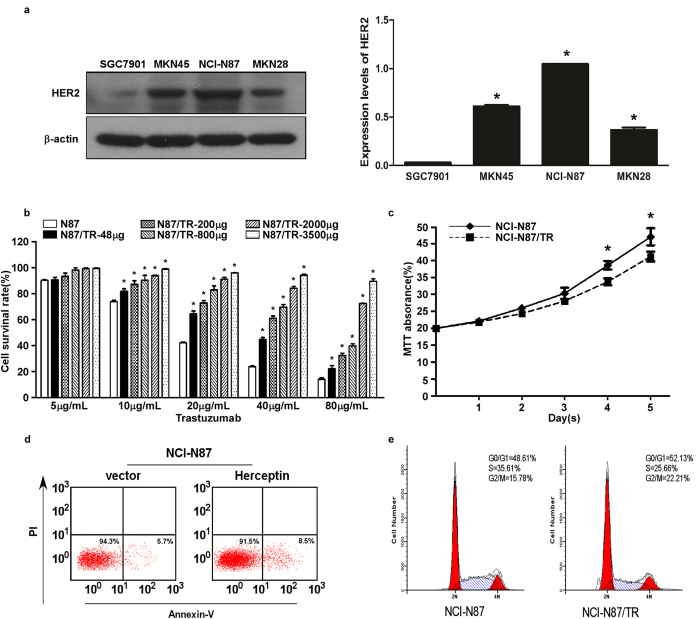
Induction of trastuzumab-resistant NCI-N87/TR cells. **a**. Western blotting showed the expression of Her-2 protein in several gastric cancer cell lines, including SGC7901, MKN45, NCI-N87, and MKN28 cells. Actin expression indicated equal loading. All the gels run under the same experimental conditions and the experiments were repeated 3 times. The representative images were cropped and shown. Data were presented as mean ± SD. *means *P* < 0.05 vs. NCI-N87. **b**. Cell survival rate of NCI-N87 cells after treatment with trastuzumab at different concentrations in the process of inducing trastuzumab-resistance. The experiments were repeated 3 times. Data were presented as mean ± SD. Control: NCI-N87 parental cells without inducing by trastuzumab. *means *P* < 0.05 vs. Control. **c**. Growth curves of NCI-N87 and NCI-N87/TR cells determined with data from the MTT assay. The experiments were repeated 3 times. Data were presented as mean ± SD. *means *P* < 0.05 vs. NCI-N87/TR. **d**. Apoptosis of NCI-N87 and NCI-N87/TR cells determined with an AV/PI Double-Dye Apoptosis Kit. **e**. Cell cycle analysis of NCI-N87 and NCI-N87/TR cells by FCM.

Expression of Her-2 protein was detected in all 4 gastric cancer cell lines (SGC7901, MKN45, NCI-N87, and MKN28) with the highest level being observed in NCI-N87 cells(*P* < 0.05) (Fig. 1a). The cell inhibiting rate of NCI-N87 cells was the largest, followed by SGC7901, MKN45, and MKN28 cells, indicating that the trastuzumab sensitivity of NCI-N87 cells was the highest (Supple 1). Therefore, we employed NCI-N87 cells for further studies because of their high Her-2 protein expression and trastuzumab sensitivity. When the concentration of trastuzumab reached 3500 μg/ml, the IC_50_ of NCI-N87 and NCI-N87/TR cells was 19.762 μg/ml and 227.523 μg/ml respectively, and the RI of NCI-N87/TR cells for trastuzumab was 11.51 (Supple 2, Fig. 1b).

Compared with parental NCI-N87 cells, NCI-N87/TR cells grew more slowly (Fig. 1c) and showed less apoptosis (91.5% vs 94.3%) (Fig. 1d). NCI-N87/TR cells displayed significant changes of the cell cycle, including an increase of cells in the G0/G1 and G2/M phases (52.13% vs. 48.61% and 22.21% vs. 15.78%) and a decrease of cells in S phase (25.66% vs. 35.61%), as well as a decline of CPI (47.87% vs. 51.39%) (Fig. 1e). The NCI-N87/TR cells also showed CDR to Taxol and DDP (RI = 2.03 and 2.69, both *P* = 0.000), while there was no resistance to 5-FU (RI = 0.97, *P* = 0.725) (Table 3).

### PTEN gene down-regulation and mutation with up-regulated phosphorylation of AKT and IGF-1R proteins in trastuzumab-resistant NCI-N87/TR cells

**Figure 2:**
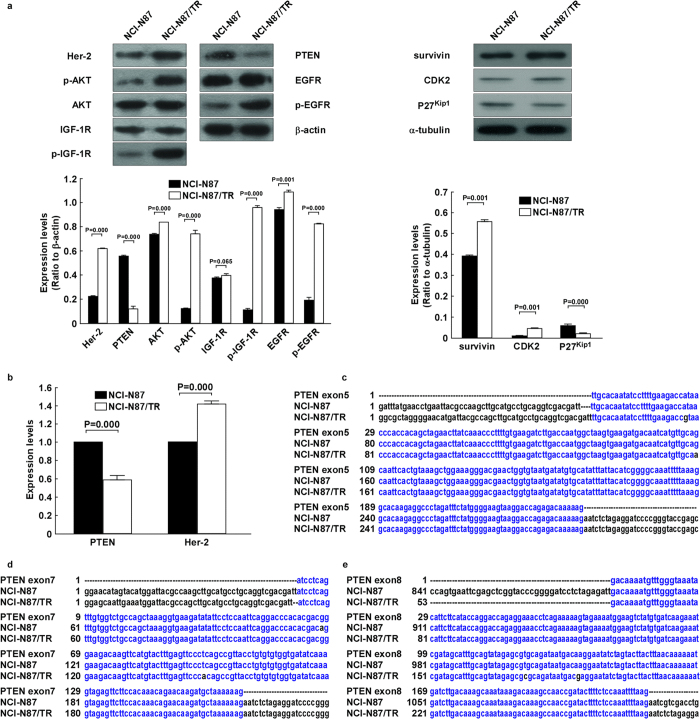
Down-regulation and mutation of the PTEN gene, as well as up-regulated phosphorylation of AKT and IGF-1R proteins, in trastuzumab-resistant NCI-N87/TR cells. **a**. Western blotting showed the expression of Her-2, PTEN, EGFR, P-EGFR, IGF-1R, P-IGF-1R, AKT, P-AKT, survivin, CDK2, and p27kipl proteins in NCI-N87 cells and NCI-N87/TR cells. Actin or tubulin expression indicated equal loading. All the gels run under the same experimental conditions and the experiments were repeated 3 times. The representative images were cropped and shown. Data were presented as mean ± SD and analyzed using Student’s t-test. **b**. Expression of the Her-2 and PTEN genes in NCI-N87 cells and NCI-N87/TR cells shown by real-time PCR. The experiments were repeated 3 times. Data were presented as mean ± SD and analyzed using Student’s t-test. **c**. Results of PTEN exon 5 sequencing. Mutations at 1309 bp and 1392 bp were detected in NCI-N87/TR cells (A→G and G→A), with the former inducing (H→R; H: His, R: Arg) and the latter inducing (A→T; A: Ala, T: Thr). **d**. Results of PTEN exon7 sequencing. A mutation at 1732 bp was found in NCI-N87 cells (G→A), inducing (R→Q; R: Arg, Q: Gln), while a mutation at 1763 bp (T→A) was silent. **e**. Results of PTEN exon 8 sequencing. Mutations at 1955 bp and 1968 bp were found in NCI-N87/TR cells (T→C and A→G), with the former being silent and the latter inducing (K→E; K: Lys, E: Glu).

Compared with NCI-N87 cells, PTEN gene and protein expression both showed a significant decrease in NCI-N87/TR cells (both *P* = 0.000) (Fig. 2a,b), along with mutation of the PTEN gene at exons 5, 7, and 8 (Fig. 2c–e). Expression of P-IGF-1R, AKT, and P-AKT proteins in NCI-N87/TR cells was markedly increased (all *P* = 0.000), while there was no change in the expression of IGF-1R protein (*P* = 0.065) (Fig. 2a).

Expression of the Her-2 gene and protein also showed a marked increase in NCI-N87/TR cells (both *P* = 0.000) (Fig. 2a,b), as did expression of EGFR, P-EGFR, survivin, and CDK2 proteins (all *P* *<* 0.001). However p27kipl protein expression was significantly decreased (*P* = 0.000) (Fig. 2a).

### PTEN gene transfection with inhibition of PI3K-AKT and IGF-1R signaling increases the trastuzumab sensitivity of NCI-N87/TR cells

**Figure 3:**
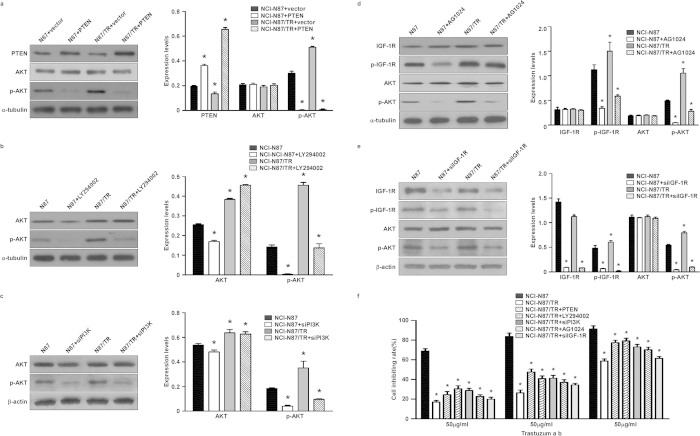
PTEN transfection or inhibition of the PI3K-AKT and IGF-1R signaling pathways increases the trastuzumab sensitivity of NCI-N87/TR cells. **a**. After PTEN gene transfection, the expression of PTEN, AKT, and P-AKT proteins was assessed by Western blotting. Tubulin expression indicated equal loading. All gels run under the same experimental conditions and the experiments were repeated 3 times. The representative images were cropped and shown. Control: cells with transfection by vector. *means *P* = 0.000 vs. Control. **b**. After treating the cells with a PI3K inhibitor (LY294002), the expression of AKT and P-AKT proteins was assessed by Western blotting. Tubulin expression indicated equal loading. All gels run under the same experimental conditions and the experiments were repeated 3 times. The representative images were cropped and shown. Control: cells without any treatment. *means *P* = 0.000 vs. Control. **c**. After transfecting the cells with PI3Kp110 siRNA, the expression of AKT and P-AKT proteins was assessed by Western blotting. Actin expression indicated equal loading. All gels run under the same experimental conditions and the experiments were repeated 3 times. The representative images were cropped and shown. Control: cells without any transfection. *means *P* = 0.000 vs. Control. **d**. After treating the cells with an IGF-1R inhibitor (AG1024), the expression of IGF-1R, P-IGF-1R, AKT, and P-AKT proteins was assessed by Western blotting. Tubulin expression indicated equal loading. All gels run under the same experimental conditions and the experiments were repeated 3 times. The representative images were cropped and shown. Control: cells without any treatment. *means *P* = 0.000 vs. Control. **e**. After transfecting the cells with IGF-1R siRNA, the expression of IGF-1R, P-IGF-1R, AKT, and P-AKT proteins was assessed by Western blotting. Actin expression indicated equal loading. All gels run under the same experimental conditions and the experiments were repeated 3 times. The representative images were cropped and shown. Control: cells without any transfection. *means *P* = 0.000 vs. Control. **f**. After PTEN gene transfection, treatment of the cells with LY294002 or AG1024, as well as siRNA targeting PI3K p110 or IGF-1R, MTT assay was performed and repeated 3 times. *means *P* < 0.05 vs. NCI-N87/TR.

Construction of the PTEN plasmid using the pBabe-puro vector yielded positive clones that had a PTEN sequence consistent with the reference sequence. The clones obtained by resistance screening were shown to have been successfully transfected with the PTEN gene. Western blotting showed that transfection with pBabe-puro-PTEN resulted in the up-regulation of PTEN protein expression and the down-regulation of P-AKT protein expression in NCI-N87 and NCI-N87/TR cells compared with transfection of the blank vector implying that PTEN gene transfection might inhibit activation of the downstream PI3K-AKT signaling pathway (both *P* = 0.000) (Fig. 3a). Drug sensitivity testing showed that after transfection with the PTEN gene, the IC_50_ of NCI-N87/TR cells decreased from 172.31 μg/ml to 100.04 μg/ml and RI decreased from 7.09 to 4.12, indicating an increase of sensitivity to trastuzumab (Table 4, Fig. 3f).

After cells were incubated with the PI3K inhibitor LY294002, as well as transfected with siRNA targeting PI3Kp110, western blotting showed that the P-AKT protein expression decreased in both NCI-N87 and NCI-N87/TR cells(both *P* = 0.000), and the descent of AKT protein phosphorylation indicating that the inhibitor LY294002 or siRNA targeting PI3Kp110 effectively blocked the PI3K-AKT signaling pathway (Fig. 3b,c). After being treated with the PI3K inhibitor LY294002 or siRNA targeting PI3Kp110, the IC_50_ of NCI-N87/TR cells decreased from 172.31 μg/ml to 97.01 μg/ml and 107.07 μg/ml, the RI decreased from 7.09 to 3.99 and 4.40 respectively, indicating an increase of sensitivity to trastuzumab (Table 4, Fig. 3f).

After cells were incubated with the IGF-1R inhibitor AG1024, as well as transfected with siRNA targeting IGF-1R, western blotting showed that the expression of P-IGF-1R and P-AKT proteins was decreased in both NCI-N87 cells and NCI-N87/TR cells(both *P* = 0.000), as was the phosphorylation of IGF-1R and AKT proteins, indicating that the inhibitor AG1024 or siRNA targeting IGF-1R effectively blocked the IGF-1R signaling pathway, and that activation of the PI3K-AKT signaling pathway was down-regulated through inhibition of the IGF-1R pathway (Fig. 3d,e). After treatment with the IGF-1R inhibitor AG1024 or siRNA targeting IGF-1R, the IC_50_ of NCI-N87/TR cells decreased from 172.31 μg/ml to 122.07 μg/ml and 148.24 μg/ml, the RI declined from 7.09 to 5.02 and 6.10 respectively, indicating an increase of sensitivity to trastuzumab (Table 4, Fig. 3f).

## Discussion

Her-2 plays an important role in the development and progression of gastric cancer. Research has shown that Her-2/neu gene expression is an independent prognostic factor for gastric cancer and that patients with over-expression of Her-2 protein have a poor prognosis and short survival time^3^,^12^.

Trastuzumab was shown to be the first biological product that prolonged the survival of metastatic gastric cancer patients^7^, so the EU and US FDA approved the use of trastuzumab combined with chemotherapy as first-line treatment for metastatic gastric cancer with Her-2 over-expression. However, the objective response rate to trastuzumab combined with chemotherapy failed to reach 50% and most patients who initially showed sensitivity to trastuzumab developed resistance within one year^7^. Therefore, investigation of resistance mechanisms and biological markers that can predict the effectiveness of trastuzumab for gastric cancer could be significant value for the clinical application of trastuzumab.

Creation of drug-resistant cell lines is the chief method of investigating multidrug resistance. Exposure to increasing doses of the target drug is frequently adopted to induce drug resistance because of the stability of the resistant cell lines thus obtained and the short experimental time, apart from manual determination of the dose increases^13^. As induction of trastuzumab-resistant human gastric carcinoma cell lines had not been previously reported in literature, the concentration regulation of trastuzumab during induction was a challenge. In the present study, NCI-N87 cells with high expression of Her-2 protein and high sensitivity to trastuzumab were exposed to increasing concentrations of trastuzumab. After 150 days, we successfully induced trastuzumab-resistant NCI-N87/TR cells when the concentration of trastuzumab in the medium had reached 3500 μg/ml, and the RI of NCI-N87/TR cells was 11.51. But unfortunately, experiments failed to induce trastuzumab resistance of MKN45 cells (Supple 3). Possible reasons might be that trastuzumab sensitivity of different gastric carcinoma cell lines was different, leading to different concentrations of trastuzumab required for induction. Therefore, future studies are needed to establish several trastuzumab-resistant gastric cancer cell lines through exploring different inducing concentrations of trastuzumab to be used for further experiments.

According to the cell growth curve, as well as examination of apoptosis and the cell cycle, the biological features of trastuzumab-resistant NCI-N87/TR cells were different from those of parental cells NCI-N87. The trastuzumab-resistant cells grew more slowly and had lower proliferative activity. In addition, the apoptotic rate was lower, the percentage of cells in G0/G1 and G2/M phases was increased, and there was a decrease of S phase cells.

To determine whether or not NCI-N87/TR cells developed CDR to chemotherapy agents, we measured the sensitivity of NCI-N87 and NCI-N87/TR cells to 5-FU, Taxol, and DDP. We found that NCI-N87/TR cells showed CDR to Taxol and DDP, but not 5-FU, indicating that there was not complete cross-resistance between trazumumab and chemotherapy agents.

PTEN (phosphates and tensin homolog deleted on chromosome ten) is a tumor suppressor gene with dual-specificity phosphatase activity. Loss of PTEN might result in continuous activation of the PI3K-AKT signaling pathway^14^,^15^. Researchs have shown the level of PTEN expression was closely associated with histologic differentiation, depth of invasion, lymph node metastasis, and prognosis in gastric cancer^16^ and the loss or down-regulation of PTEN in 58.7% gastric cancer patients and an association with advanced clinical stage^17^.

The relationship between trastuzumab resistance and the PTEN gene and PI3K-AKT signaling pathways was mainly focused on breast cancer^9^,^18^,^19^, so whether similar resistance mechanisms operate in gastric cancer has been unclear. Therefore, we examined expression of the PTEN gene and PTEN, AKT and P-AKT proteins in NCI-N87 and NCI-N87/TR cells. We showed that PTEN expression was decreased significantly in trastuzumab-resistant NCI-N87/TR cells at both the gene and protein level, and that phosphorylation of AKT protein was up-regulated, indicating that PTEN down-regulation along with activation of the PI3K-AKT signaling pathways might be one of the major resistance mechanisms of gastric cancer to trastuzumab.

Gene mutation is the main reason for loss of PTEN activity. Lima *et al.*^20^ used PCR-SSCP to examine 48 samples of gastric adenocarcinoma, and only one had a translocation mutation. However, Wang *et al.*^21^ studied 60 samples of progressive gastric cancer and found 17 (28.3%) with PTEN gene mutation. We also sequenced the PTEN gene in NCI-N87 and NCI-N87/TR cells, and the results showed that there were PTEN mutation at exons 5, 7, and 8, indicating that PTEN mutation might played an important role in the resistance of gastric cancer to trastuzumab. Here we described a novel observation that a mutation at 1732 bp in exon 7 was found in NCI-N87 cells (G→A), while the mutation absented and backed to normal in NCI-N87/TR cells. There was no report that PTEN gene mutation happened in NCI-N87 cells and our research found the phenomenon for the first time. The more interest was that this mutation returned to normal in the process of inducing trastuzumab resistance. It might be attributed to the instability of genome in cancer cells. The results further revealed that PTEN gene mutation took part in regulating the trastuzumab resistance in gastric cancer.

IGF-1R is a member of the tyrosine kinase receptor family, and activation of IGF-IR tyrosine kinase induces the transduction of signaling pathways that include Ras-Raf-MAPK and PI3K-AKT. Over-expression of IGF-IR and its ligand is common in gastric cancer, and the serum concentration of GH or IGF-I is often increased^22^. It was still not very clear whether the over-expression of IGF-IR predicted resistance to trastuzumab^23^,^24^. We examined expression of the IGF-1R and P-IGF-1R proteins in NCI-N87 and NCI-N87/TR cells. We showed that phosphorylation of IGF-1R proteins was up-regulated, indicating that activation of the IGF-1R signaling pathways might be involved in the resistance of gastric cancer to trastuzumab.

In order to confirm the above mechanisms in gastric cancer, we studied on the PTEN gene transfection, inhibition of the PI3K-AKT and IGF-1R signaling pathways. We used pBabe-puro-PTEN to transfect trastuzumab-resistant NCI-N87/TR cells with low PTEN expression, in order to up-regulate PTEN gene expression. After transfection, the expression of PTEN protein increased in NCI-N87/TR cells while phosphorylation of AKT protein decreased, and tumor cell sensitivity to trastuzumab also increased. This indicated that pBabe-puro-PTEN transfection was able to up-regulate PTEN gene expression in trastuzumab-resistant NCI-N87/TR cells and inhibit activation of the PI3K-AKT signaling pathway, thus reversing the trastuzumab resistance of gastric cancer cells. We also treated trastuzumab-resistant NCI-N87/TR cells with a PI3K inhibitor (LY294002) or an IGF-1R inhibitor (AG1024), as well as siRNA targeting PI3K p110 or IGF-1R , and the results showed that the phosphorylation of both AKT and IGF-1R proteins decreased in the treated cells, while sensitivity to trastuzumab increased.

Multiple factors influenced the resistance to molecular-targeting drugs. The major purpose of our manuscript was to study the regulation of trastuzumab resistance in gastric cancer by the PTEN gene, downstream AKT, and bypass IGF-IR signaling pathway. Among the PTEN gene transfection, inhibition of the PI3K-AKT and IGF-1R signaling pathways, the increased degree of NCI-N87/TR cells sensitivity to trastuzumab was the highest when treated with PI3K inhibitor LY294002. Accordingly, these results confirmed that trastuzumab resistance of NCI-N87/TR cells was related to the PI3K-AKT signaling pathway which was activated by PTEN gene down-regulation/mutation and over-activity of IGF-1R signaling pathway.

Based on these results, we concluded that activation of the PI3K-AKT signaling pathway was one of the major mechanisms leading to resistance of NCI-N87/TR gastric cancer cells to trastuzumab, which was probably associated with PTEN gene down-regulation and mutation, as well as with over-activity of the IGF-1R signaling pathway. Thus, the PTEN gene might be a factor in the trastuzumab resistance of gastric cancer, and its downstream PI3K-AKT and bypass IGF-1R signaling pathways might be new targets for clinical research on drug resistance in the future.

Trastuzumab inhibited cell growth by antagonizing the Her-2 signal transduction pathway. It was still not clear whether the up-expression of Her-2 protein induced resistance to trastuzumab. Interestingly, it had been reported that higher levels of Her-2 localized in mitochondria were more resistant to trastuzumab in breast cancer cells^25^. There had been other reports that co-expression of Her-2 and EGFR proteins with mutual reinforcement in breast cancer cells could inhibit apoptosis by up-regulating the expression of survivin and were related to trastuzumab sensitivity^26^,^27^. In our study, we found the up-regulation of Her-2, EGFR, P-EGFR and survivin proteins, along with a decrease of apoptosis in NCI-N87/TR cells, indicating that co-overexpression of Her-2 and EGFR might participate in regulating the resistance of gastric cancer to trastuzumab through up-regulating the expression of survivin protein. We also found the up-regulation of CDK2 protein along with down-regulation of p27kipl protein in NCI-N87/TR cells. There had been reports that the down-regulation of p27kip1 protein caused by the activation of CDK2 protein was related to trastuzumab resistance of breast cancer^28^,^29^, indicating it might be one another mechanism involved in the trastuzumab resistance in gastric cancer . All of these issues need further investigation in the future.

## Additional Information

**How to cite this article**: Zuo, Q. *et al.* Development of trastuzumab-resistant human gastric carcinoma cell lines and mechanisms of drug resistance. *Sci. Rep.* **5**, 11634; doi: 10.1038/srep11634 (2015).

## Supplementary Material

Supplementary Information

## Figures and Tables

**Table 1:** Primer sequences for Real-time PCR

**Gene name**	**Gene name**	**Primer sequence (5′ to 3′)**	**Primer length (bp)**	**Amplified length (bp)**
β-actin	β-actin-F	TGGCACCCAGCACAATGAA	20	186
	β-actin-R	CTAAGTCATAGTCCGCCTAGAAGCA	25	
PTEN	PTEN-F	GGCGGAACTTGCAATCCTC	20	234
	PTEN-R	TTCCTCTGGTCCTGGTATG	20	
Her-2	Her-2-F	GAAGCCTCACAGAGATCTTG	20	174
	Her-2-R	CCTTACACATCGGAGAACAG	20	

**Table 2:** Primer sequences for gene sequencing

**Gene name**	**Gene name**	**Primer sequence(5′ to 3′)**	**Primer length (bp)**	**Amplified length (bp)**
PTEN- Exon5	Exon5-F	TTGCACAATATCCTTTTGAAGAC	23	239
	Exon5-R	CTTTTTGTCTCTGGTCCTTAC	22	
PTEN- Exon7	Exon7-F	ATCCTCAGTTTGTGGTCTGCC	21	167
	Exon7-R	CTTTTTTAGCATCTTGTTCTGTTTG	22	
PTEN- Exon8	Exon8-F	GACAAAATGTTTCACTTTTGGG	22	225
	Exon8-R	CTTAAAATTTGGAGAAAAGTATCGG	25	

**Table 3:** Sensitivity and cross drug resistance of NCI-N87 and NCI-N87/TR cells to chemotherapeutic drugs (mean ± SD)

**Drug**	**IC50(μg/ml)**		**RI**
	**NCI-N87**	**NCI-N87/TR**	
5-FU	0.25 ± 0.02	0.24 ± 0.03	0.97
Taxol	2.63 ± 0.08	5.34 ± 0.16*	2.03
DDP	0.16 ± 0.01	0.42 ± 0.03*	2.69

^*^P = 0.000 vs. NCI-N87

**Table 4:** IC50 and RI of different cells after treatment with trastuzumab

**Cell**	**IC50(μg/ml)**	**RI**
NCI-N87	24.31	
NCI-N87/TR	172.31	7.09
NCI-N87/TR + PTEN	100.04	4.12
NCI-N87/TR + LY294002	97.01	3.99
NCI-N87/TR + siPI3K	107.07	4.40
NCI-N87/TR + AG1024	122.07	5.02
NCI-N87/TR + siIGF-1R	148.24	6.10
