# Supplementary material for: Development of trastuzumab-resistant human gastric carcinoma cell lines and mechanisms of drug resistance
Source: Sci Rep. 2015 Jun 25;5:11634. doi: 10.1038/srep11634 (PMC4479993; doi:10.1038/srep11634)
Supplement: Supplementary Information [file srep11634-s1.doc]

**Development of trastuzumab-resistant human gastric carcinoma cell lines and mechanisms of drug resistance**

Qiang Zuo ^†^, Jing Liu ^†^, Jingwen Zhang, Mengwan Wu, Lihong Guo, and Wangjun Liao^*^

**Supplemental Table 1 Cell inhibiting rate (%) of different gastric cancer cells after treatment with trastuzumab (mean±SD)**

| Trastuzumab (μg/ml) | SGC7901 | MKN45 | NCI-N87 | MKN28 |
| --- | --- | --- | --- | --- |
| 0 | 0 | 0 | 0 | 0 |
| 0.5 | 42.68±2.79 | 42.89±2.43 | 43.38±3.03 | 41.94±0.77 |
| 1 | 45.05±4.37 | 45.99±5.28 | 44.88±0.44 | 45.95±0.71 |
| 5 | 64.38±1.05 | 59.48±3.83 | 62.09±0.53 | 57.23±1.46 |
| 10 | 72.52±2.31 | 63.35±2.98* | 74.27±1.74 | 62.22±1.31* |
| 20 | 78.00±1.83* | 67.81±2.64* | 83.85±1.84 | 66.60±1.44* |
| 40 | 81.19±0.85* | 68.87±2.46* | 84.29±0.50 | 69.91±0.24* |

**P*＜0.05 vs. NCI-N87

**Supplemental Table 2 IC50 and RI of NCI-N87 cells after treatment with trastuzumab at different inducing concentrations**

| Trastuzumab (μg/ml) | IC50 (μg/ml) | RI |
| --- | --- | --- |
| 0 | 19.762 |  |
| 48 | 32.023 | 1.62 |
| 96 | 35.579 | 1.80 |
| 200 | 48.372 | 2.45 |
| 400 | 54.979 | 2.78 |
| 800 | 61.887 | 3.13 |
| 1500 | 140.702 | 7.12 |
| 2000 | 147.424 | 7.46 |
| 3000 | 186.347 | 9.43 |
| 3500 | 227.523 | 11.51 |

**Supplemental Table 3** Cell survival rates (%) of MKN45 cells after treatment with trastuzumab at different concentrations (mean±SD).

| Cell | Trastuzumab (μg/ml) | | | | | |
| --- | --- | --- | --- | --- | --- | --- |
|  | 0 | 5 | 10 | 20 | 40 | 80 |
| MKN45 | 100 | 77.43±2.12 | 60.16±1.39 | 30.02±1.01 | 16.85±1.03 | 11.55±0.98 |
| MKN45/TR48μg/ml | 100 | 80.89±2.31 | 63.49±1.18 | 35.08±1.17 | 20.47±0.98 | 15.12±0.79 |
| MKN45/TR96μg/ml | 100 | 82.05±1.43 | 64.87±1.21 | 37.04±1.31 | 22.52±1.03 | 18.03±0.96 |
| MKN45/TR200μg/ml | 100 | 84.14±2.11 | 65.28±1.41 | 37.62±1.01 | 22.80±0.76 | 17.66±1.12 |
| MKN45/TR400μg/ml | 100 | 83.57±1.72 | 65.58±1.51 | 37.66±1.12 | 22.76±1.14 | 19.36±0.91 |
| MKN45/TR800μg/ml | 100 | 82.59±1.89 | 66.46±1.43 | 39.23±1.27 | 24.06±1.03 | 19.50±0.87 |
